# Supplementary material for: Higher-order structure and proteoforms of co-occurring C4b-binding protein assemblies in human serum
Source: EMBO J. 2024 May 29;43(14):10. doi: 10.1038/s44318-024-00128-y (PMC11251186; doi:10.1038/s44318-024-00128-y)
Supplement: Supplementary file 1 — Appendix [file 44318_2024_128_MOESM1_ESM.pdf]

Appendix

Higher-order structure and proteoforms of co-occurring C4b-binding protein assemblies in human serum

Tereza Kadavá, Johannes F. Hevler, Sofia Kalaidopoulou Nteak, Victor C. Yin, Juergen Strasser, Johannes Preiner, and Albert J.R. Heck*

Table of content

Appendix Figure S1. 2
Appendix Figure S2. 3
Appendix Figure S3. 4
Appendix Figure S4. 5
Appendix Figure S5. 6

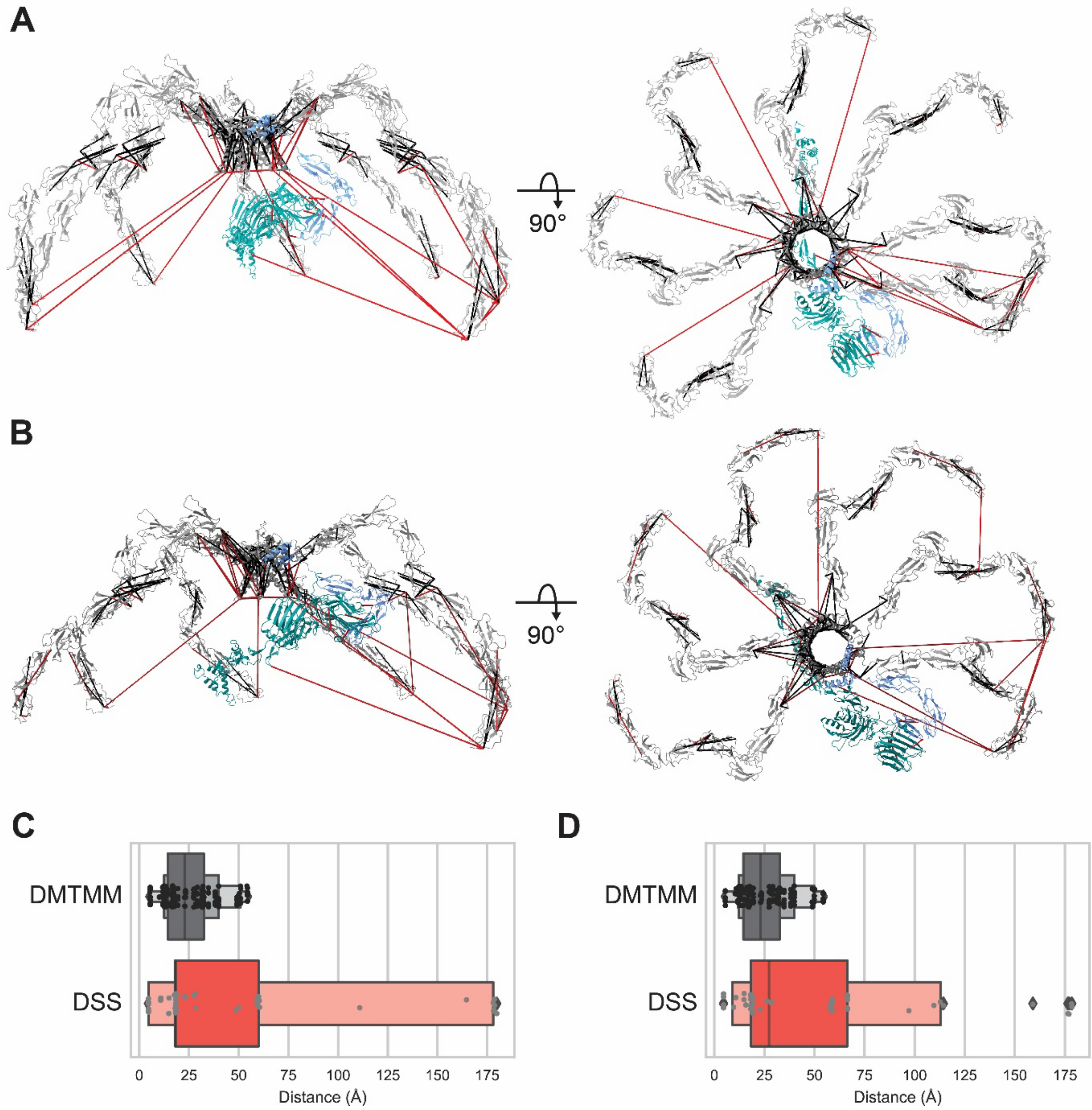

Appendix Figure S1. Full-length structural models of C4BP(β+) with XL-MS restraints and distance plots. Visualized were the shortest cross-links with 2Å difference allowed, DSS restraints are shown in red, and DMTMM in black. (**A**), (**C**) α7β1 + ProS model and respective distance plot. (**B**), (**D**) α6β1 + ProS model and respective distance plot. All distance plots are displaying ~25 Å median values corresponding to the expected values, yet in the DSS sample we also observe over-length cross-links, corresponding predominantly to CCP1 involving restraints. Models and graphs were generated in ChimeraX using the XMAS bundle.

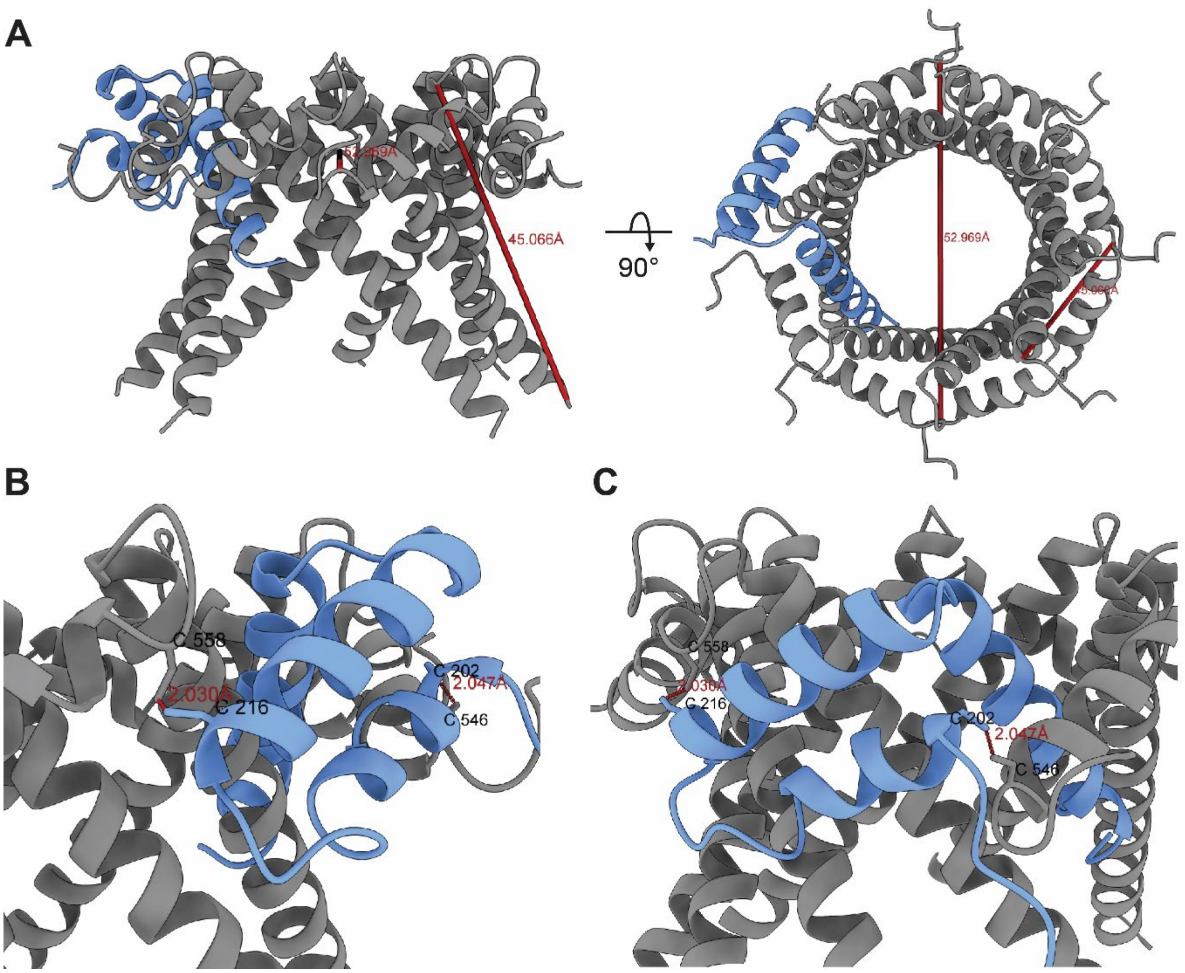

Appendix Figure S2. Dimensions of the C4BP oligomerization core, and disulfides connecting C4BPα and C4BPβ.

A. Structural model of αβ1 with diameter of 53 Å (distance between C4BPα P562 and L597) and thickness of 4.5 Å (distance between opposite C4BPα C546).

B. Disulfide bridge C4BPα(C558)- C4BPβ(C216). C) Disulfide bridge C4BPα(C202)- C4BPβ(C546).

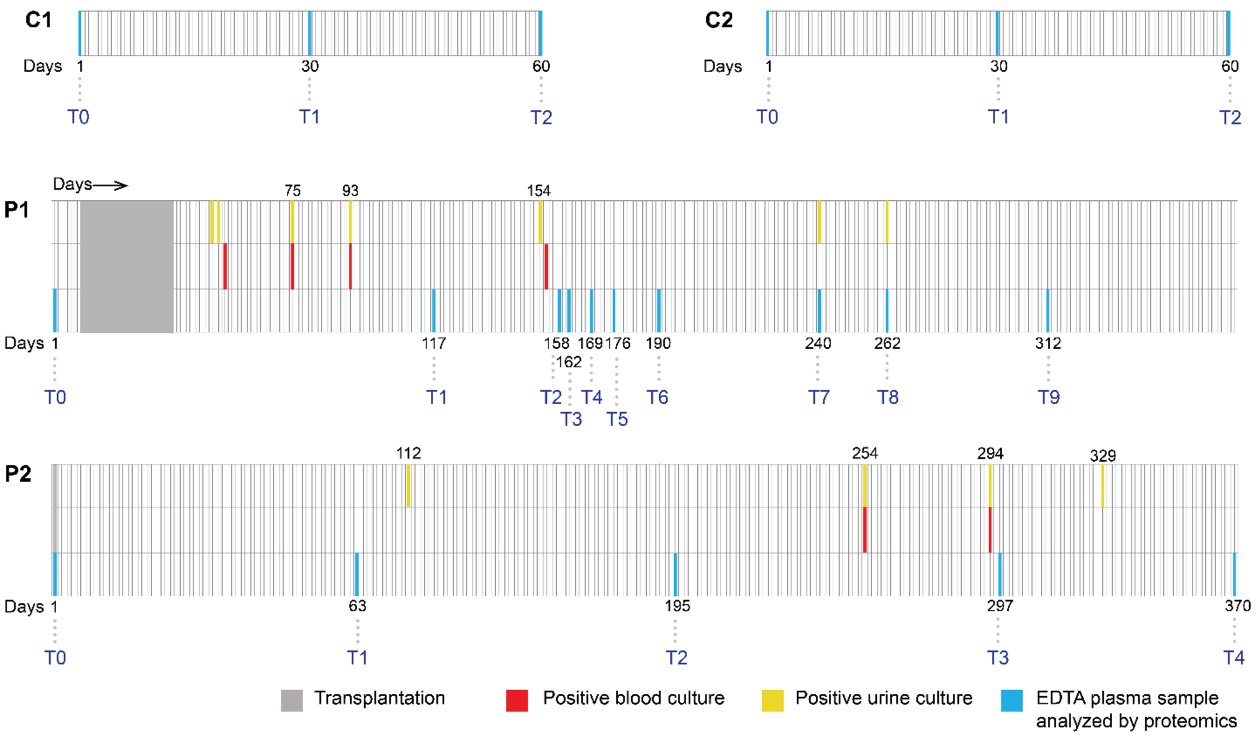

Appendix Figure S3. Overview of patients (P1, P2) and healthy controls (C1, C2) sampling for plasma proteomics. Figure shows timeline of EDTA plasma samples analyzed by proteomics (blue) and confirmed acute phase inflammation by either positive blood (red) or urine (yellow) culture.

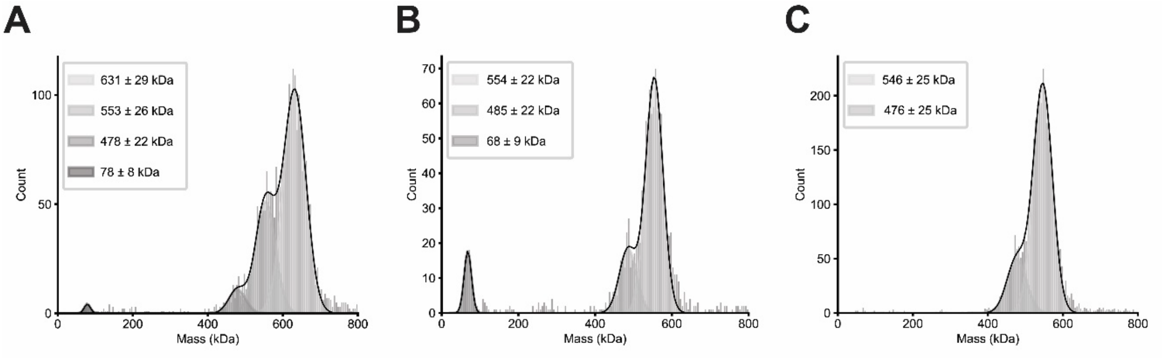

Appendix Figure S4. Comparison of FA treated C4BP and C4BPΔProS. Figure shows MP histograms for (**A**) native C4BP (PBS, pH = 7.4), (**B**) FA treated C4BP (PBS, pH ~ 2), (**C**) C4BPΔProS as analyzed by HS-AFM (PBS, pH = 7.4).

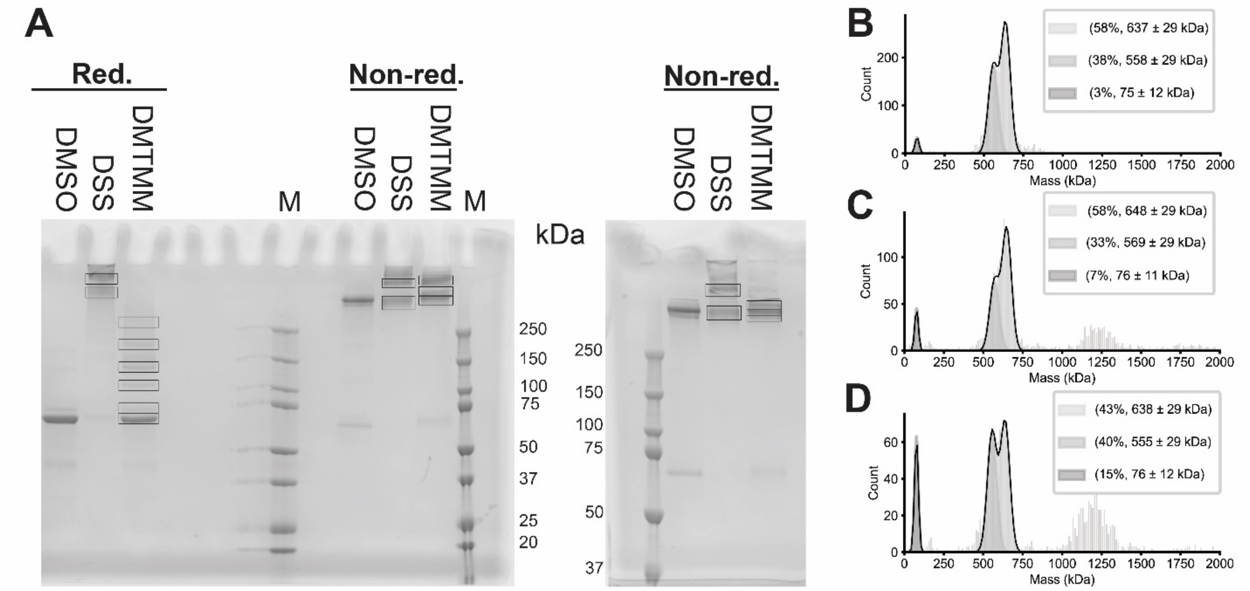

Appendix Figure S5. MP and SDS-PAGE of cross-linked C4BP. (**A**) Reducing (Red.) or non-reducing (Non-red.) SDS-PAGE of C4BP samples: DMSO – C4BP in 5% DMSO, DSS – C4BP cross-linked by 1 mM DSS, DMTMM - C4BP cross-linked by 5 mM DMTMM. Black rectangles correspond to excised bands (labeled from 1 corresponding to the top band for each line respectively). Precision Plus Protein Dual Color (Biorad) was used as a marker. Figure subplots (**B**-**D**) show MP results of C4BP: (**B**) incubated in 5% DMSO, (**C**) DSS cross-linked, (**D**) DMTMM cross-linked.
