# Supplementary material for: Higher-order structure and proteoforms of co-occurring C4b-binding protein assemblies in human serum
Source: EMBO J. 2024 May 29;43(14):10. doi: 10.1038/s44318-024-00128-y (PMC11251186; doi:10.1038/s44318-024-00128-y)
Supplement: Supplementary file 3 — Movie EV1 [file 44318_2024_128_MOESM3_ESM.zip › Movie_EV1_caption.docx]

Movie EV1. The flexibility of α7β1+ProS in strong immobilization buffer. α7β1+ProS on freshly cleaved mica incubated with C4BP (6 µg/mL in a weak immobilization buffer, 5 min) in a strong immobilization buffer, recorded at 1000 ms/frame. Scan size: 100 x 100 nm^2^ (200x200 pixel); Cropped to 65 x 80 nm^2^. Color scale range 4.6 nm. Playback at 1x recording speed.
