# Supplementary material for: Higher-order structure and proteoforms of co-occurring C4b-binding protein assemblies in human serum
Source: EMBO J. 2024 May 29;43(14):10. doi: 10.1038/s44318-024-00128-y (PMC11251186; doi:10.1038/s44318-024-00128-y)
Supplement: Supplementary file 5 — Movie EV3 [file 44318_2024_128_MOESM5_ESM.zip › Movie_EV3_caption.docx]

Movie EV3. ProS is attached to C4BPβ non-covalently. Freshly cleaved mica incubated with C4BP (6 µg/mL in weak immobilization buffer, 5 min) imaged in a weak immobilization buffer, recorded at 510 ms/frame. ProS fully dissociates after four seconds. Scan size: 200 x 200 nm^2^ (200x200 pixel); Cropped to 155 x 145 nm^2^. Color scale 5.4 nm. Playback at 1x recording speed.
