# Supplementary material for: Higher-order structure and proteoforms of co-occurring C4b-binding protein assemblies in human serum
Source: EMBO J. 2024 May 29;43(14):10. doi: 10.1038/s44318-024-00128-y (PMC11251186; doi:10.1038/s44318-024-00128-y)
Supplement: Supplementary file 13 — Expanded View Figures [file 44318_2024_128_MOESM13_ESM.pdf]

Expanded View Figures

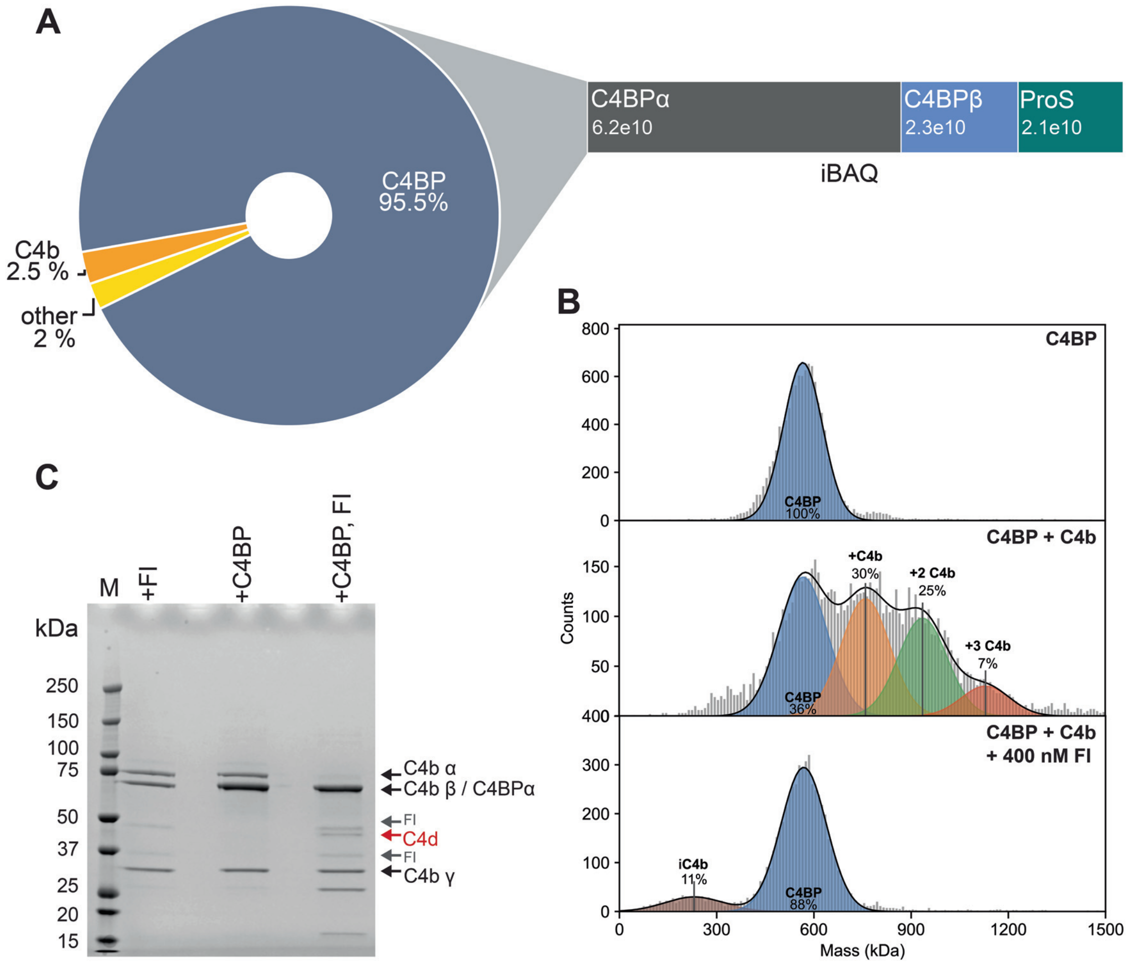

Figure EV1. C4BP sample composition and activity.

(**A**) Composition of the commercial C4BP sample accessed by bottom-up proteomics. The figure shows an abundance of non-contaminant proteins based on their intensity Based Absolute Quantitation (iBAQ) values and suggests that more than 95% of the sample is composed of C4BP HOS formed by C4BPα, C4BPβ interacting with ProS. (**B**) Mass photometry results highlight C4BP interaction with C4b. The figure shows mass distributions of C4BP (upper panel), C4BP, and C4b in a 1:1 mass ratio (middle panel), and C4BP and C4b in a 1:1 mass ratio with 400 nM FI (bottom panel). The results affirmed C4BPα mediated interaction (Dahlbäck et al, 1983; Blom et al, 2001) resulting in multiple C4b molecules bound to one C4BP. Further, the results show that FI addition leads to C4b degradation (iC4b) and complex disruption. (**C**) FI-mediated cleavage of C4b in the presence of C4BP. The figure shows the reducing SDS-PAGE of C4b with FI, C4BP, and C4b in a 1:1 mass ratio, and C4BP and C4b in a 1:1 mass ratio with FI, from left to right respectively. The latter shows cleavage of the C4b α-chain by FI in the presence of C4BP. The other two samples display no C4b α-chain showing that C4BP is an essential cofactor in the C4b α-chain degradation. The bands were assigned based on (Blom et al, 2003b). Source data are available online for this figure.

 © The Author(s)

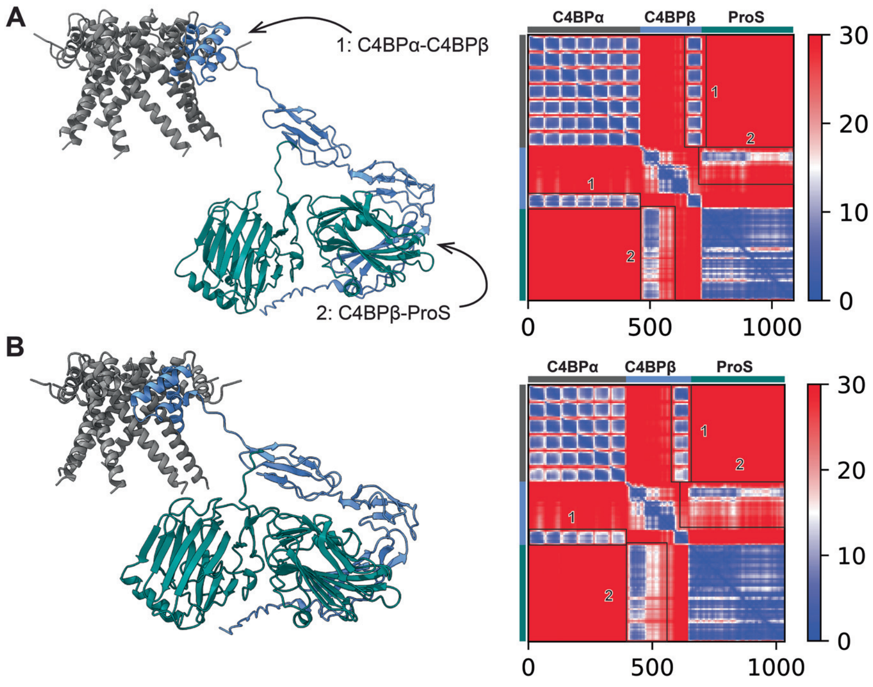

Figure EV2. Structural models of C4BP core.

The figure shows models of the C4BP core generated by AlphaFold-Multimer (v. 2.2.0) and PAE plots for (**A**) α7β1+ProS and (**B**) α6β1+ProS variants. The models show the insertion of C4BPβ into the C4BPα core (area 1 in PAE plot) and the interaction of C4BPβ-ProS (area 2 in PAE plot). The models cover C4BPα (541–597), C4BPβ (1–252), and ProS (284–676). Source data are available online for this figure.

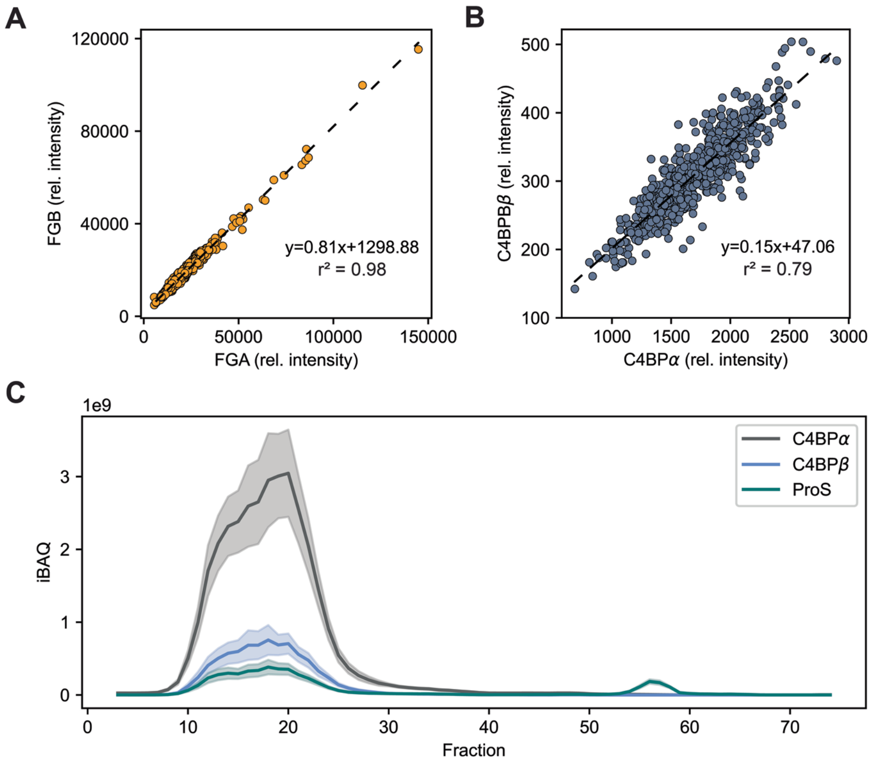

Figure EV3. C4BP in human serum.

(**A**) Correlation of FGA and FGB in a dataset of ~670 human plasma samples (Demichev et al, 2021). The figure shows relative intensities as identified and quantified by DIA-MS proteomics. FGA and FGB relative intensities are shown on the x and y-axis, respectively. The proteins are known to form a stable complex in a 1:1 (Kollman et al, 2009) ratio, which is confirmed by the observed correlation. (**B**) Correlation of C4BPα and C4BPβ in a dataset of ~670 human plasma samples (Demichev et al, 2021). The figure shows relative intensities as identified and quantified by DIA-MS proteomics. Relative intensities of C4BPα and C4BPβ are shown on the x and y-axis, respectively. The figure shows a correlation of C4BPα and C4BPβ relative intensities and indicates more variability in the C4BP assembly compared to the FGA and FGB (**A**). (**C**) Elution profile of C4BPα (gray), C4BPβ (blue), and ProS (teal) in serum SEC. The figure shows pooled healthy donor serum SEC-LC-MS in an experimental triplicate with a standard deviation interval. Fraction numbers are shown on the x-axis and sum iBAQ values are plotted on the y-axis. The results show co-elution of C4BPα, C4BPβ, and ProS in high MW fractions (8–30) affirming their organization in HOS. The low MW fractions (50–60) display elution of unbound ProS supporting the observations in Fig. 7. Source data are available online for this figure.

 © The Author(s)
